# Calculation of Toric Intraocular Lens Power with the Barrett Calculator and Data from Three Keratometers

**DOI:** 10.1155/2021/7712345

**Published:** 2021-08-20

**Authors:** Jing Dong, Yaqin Zhang, Xiaogang Wang

**Affiliations:** ^1^The First Hospital of Shanxi Medical University, Taiyuan, Shanxi, China; ^2^Shanxi Eye Hospital Affiliated to Shanxi Medical University, Taiyuan, Shanxi, China

## Abstract

**Aim:**

To investigate the interdevice agreement for differences in toric power calculated using data on anterior corneal astigmatism obtained with corneal topography/ray-tracing aberrometry (iTrace), partial coherence interferometry (IOLMaster 500), and Scheimpflug imaging (Pentacam).

**Methods:**

The analysis included 101 eyes (101 subjects) with regular astigmatism. The main outcome measures were corneal cylinder power, axis of astigmatism, and keratometry values. Toricity and toric IOL power were calculated using the online Barrett toric calculator. Interdevice agreement for measurement and calculation was assessed using a paired sample *t*-test and a nonparametric test.

**Results:**

Significant interdevice differences were noted in the magnitude of astigmatism and flat, steep, and mean keratometry values between iTrace and IOLMaster (all *P* < 0.01); in flat, steep, and mean keratometry values (all *P* < 0.001) but not in the magnitude of astigmatism (*P*=0.325) between iTrace and Pentacam; and in the magnitude of astigmatism and steep and mean keratometry values (all *P* < 0.01) but not in flat keratometry values (*P*=0.310) between IOLMaster and Pentacam. The toric IOL power calculated using data from the three devices showed the following trend: iTrace > IOLMaster (0.49 ± 0.36, *P* < 0.001) and Pentacam (0.39 ± 0.42, *P* < 0.001) and Pentacam was <IOLMaster (−0.10 ± 0.39, *P*=0.009). There were differences in toricity calculated using data from the three devices (*P*=0.004).

**Conclusions:**

Differences in toric IOL power and toricity calculated using anterior keratometry data from iTrace, IOLMaster 500, and Pentacam should be noted in clinical practice.

## 1. Introduction

Because postoperative visual quality of patients with cataract is affected by both surgically induced astigmatism (SIA) and preoperative corneal astigmatism, the use of optimal size and location for clear corneal incision and measurement of precise preoperative corneal astigmatism should both be considered for improving postoperative visual quality in patients undergoing cataract surgery [1–3]. Rational surgical planning (toric intraocular lens (IOL), clear corneal incision at the steepest axis, peripheral corneal relaxing incisions, etc.) plays an important role in astigmatism correction. Currently, there is no standard device for measuring corneal astigmatism and calculating toric IOL in a clinical setting. Different types of keratometers (ray-tracing aberrometry, partial coherence interferometry, and Scheimpflug imaging system) may provide different corneal astigmatism values for the same eye, which might provide different toricity choices for toric IOLs, even with use of the same formula.

iTrace ray-tracing aberrometry (Tracey^TM^ Technologies, Texas, USA), on the basis of corneal topography, can provide simulated keratometry (SimK) and astigmatism data with a 3.0 mm-diameter ring centered on the anterior corneal apex [4]. IOLMaster 500 (Carl Zeiss Meditec, Germany) measures anterior corneal astigmatism and curvature by analyzing the real position of each pair of reflection spots (six spots of light arranged in a hexagonal pattern) from the anterior surface of the cornea with an approximately 2.3–2.5 mm-diameter ring [5]. The Pentacam Scheimpflug imaging system (OCULUS, Wetzlar, Germany) can capture 25–50 images by rotating 360° in one examination. Furthermore, it can image and perform automated measurement of the anterior and posterior corneal surfaces. Anterior corneal astigmatism data, as computerized values focused on the anterior 3.0 mm-diameter region of the cornea, can be centered on the corneal apex or pupil [6]. Considering that iTrace and IOLMaster 500 measure corneal curvature using data centered on the apex, the Pentacam-measured anterior axial keratometry data on the 3 mm-diameter ring centered on the corneal apex were used in this study.

Many toric IOL calculation methods have been reported, but studies demonstrated that the Barrett toric calculator showed better performance than did the other calculators [7]. Moreover, a recent study demonstrated that astigmatism prediction errors with and without posterior corneal curvature measured and calculated using the updated Barrett toric calculator showed similar results [8].

Considering that iTrace, IOLMaster, and Pentacam are commonly used keratometry devices and Barrett toric calculator is a relatively good toricity calculator in a clinical setting, we aimed to compare the differences in toricity calculated on the basis of the data obtained by the aforementioned three devices and the Barrett toric calculator in this study.

## 2. Methods

### 2.1. Subjects

This cross-sectional observational study was performed at Shanxi Eye Hospital. The research protocol was approved by the institutional review board of Shanxi Eye Hospital and carried out according to the tenets of the Declaration of Helsinki. Written informed consent was obtained from each subject after explaining the nature of this study.

Consecutive patients were enrolled between April 2017 and January 2019. The inclusion criteria were as follows: presence of corneal regular astigmatism and absence of systemic diseases, pathological alteration of the anterior segment (such as dry eye [9], keratoconus, zonular dialysis, pseudoexfoliation syndrome, or corneal opacity), retinal diseases impairing visual function, and previous anterior or posterior segment surgery. Because of unstable fixation during each examination, patients who failed to cooperate with any data acquisition process were excluded from this study, as described in a previous study [10].

### 2.2. Data Acquisition

Corneal keratometry data were obtained using iTrace, IOLMaster 500, and Pentacam HR, in the same sequence, for each eye. Good-quality measurements were used in the final analysis. For iTrace, IOLMaster 500, and Pentacam HR, software versions 6.1.0, 7.5, and 1.20r36, respectively, were used. All measurements were performed in a semidark room. The subjects were asked to place their chin on the chin rest and press the forehead against the forehead strap. The eye was then aligned to the visual axis by using a central fixation light or target. The subjects were instructed to perform a complete blink before each measurement. A single trained operator (YQZ) performed all the examinations using the three devices.

### 2.3. IOL Power and Toricity Calculation

IOL power and toricity were calculated using the online Barrett toric calculator v2.0 (https://www.apacrs.org/disclaimer.asp?info=3). Axial length (AL) and the optical anterior chamber depth (ACD) measured by IOLMaster 500 were used as biometry data for calculation. The 3 mm SimK data obtained using iTrace and axial keratometry data of the 3 mm ring centered on the anterior corneal apex obtained using Pentacam HR were used for calculation. Moreover, target refraction was set as plano, and incision SIA and incision location were set as 0.5 D and 120° for each calculation. Alcon SN6ATx IOL model with a lens factor of 2.02 and *A* constant of 119.26 were used for each calculation.

### 2.4. Double-Angle Plots of Astigmatism

We used the double-angle plot method, which was described by Abulafia et al., to plot the astigmatism data of each device [11]. Unlike single-angle plots, it can display the magnitude and axis of the mean astigmatism and the confidence ellipse, which is helpful for the qualitative assessment.

### 2.5. Statistics

Statistical analyses were performed using commercial software (SPSS for Windows, version 13.0; SPSS Inc., Illinois, USA). The Kolmogorov–Smirnov test was used to assess data normality. The statistical significance of the interdevice difference (magnitude astigmatism, keratometry values, and IOL power) was investigated using the paired two-tailed *t*-test. For Bonferroni correction for multiple comparisons, all tests had a significance level of 2.5%. Friedman test, a nonparametric test, was utilized to compare the astigmatism axis, toricity, and toric IOL axis among the three devices. All tests had a significant level of 5%.

## 3. Results

In this study, 101 eyes of 101 subjects were finally included. Demographics of the study population are summarized in Table 1.

**Figure 1:**
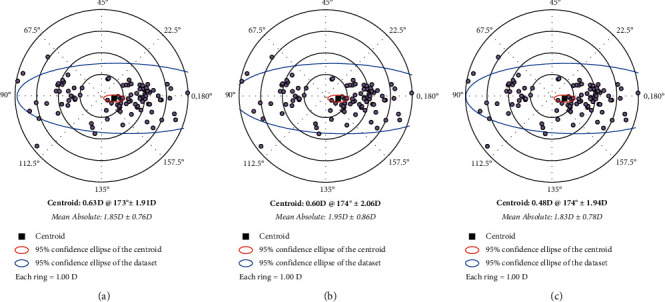
Double-angle plots of differences in anterior corneal astigmatism measured by iTrace (a), IOLMaster 500 (b), and Pentacam (c). Note: centroid (solid square); 95% confidence of the ellipse of the centroid (red color); 95% confidence ellipse of the dataset (blue color); each ring = 1.0 D.

The mean corneal SimK values and mean difference between parameters obtained using iTrace, IOLMaster 500, and Pentacam HR are listed in Tables 2 and 3 and Figure 1. No significant differences in corneal astigmatism axis values obtained using the three devices were noted (*P*=0.967).

As demonstrated by Table 3, significant interdevice differences existed between iTrace and IOLMaster for the magnitude of astigmatism and for flat, steep, and mean keratometry values (all *P* < 0.01); between iTrace and Pentacam for flat, steep, and mean keratometry values (all *P* < 0.001) but not for the magnitude of astigmatism (*P*=0.325); and between IOLMaster and Pentacam for the magnitude of astigmatism and steep and mean keratometry values (all *P* < 0.01) but not for flat keratometry values (*P*=0.310).

**Figure 2:**
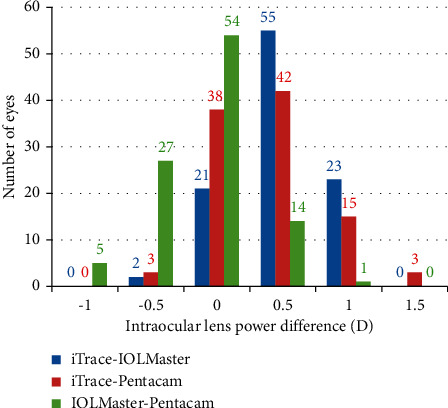
Participants whose intraocular lens power difference changed from −1.0 to 1.5 D as calculated using data obtained by iTrace, IOLMaster, and Pentacam.

As demonstrated by Table 4, the IOL power calculated using data obtained by iTrace was significantly higher than that by IOLMaster (0.49 ± 0.36, *P* < 0.001) and Pentacam (0.39 ± 0.42, *P* < 0.001), and that calculated using data obtained by Pentacam was significantly lower than that by IOLMaster (−0.10 ± 0.39, *P*=0.009). With regard to IOL power comparison among participants, 23 (22.8%), 18 (17.8%), and 6 (5.9%) eyes demonstrated an IOL power difference ≥1.0 D between iTrace and IOLMaster, between iTrace and Pentacam, and between IOLMaster and Pentacam, respectively (Figure 2).

**Figure 3:**
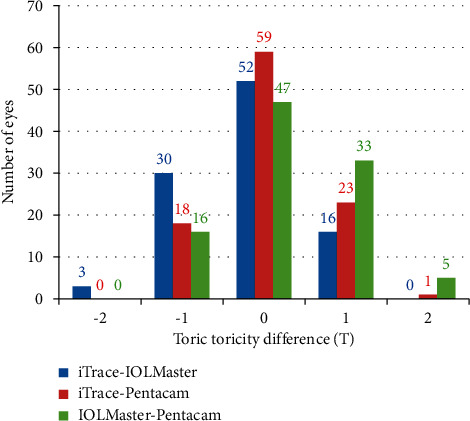
Participants whose toricity difference changed by ≤2 as calculated using astigmatism data obtained by iTrace, IOLMaster, and Pentacam.

Friedman test showed that a difference in toricity calculation existed among the three groups (*P*=0.004). However, the toric axis showed no significant difference among the three groups (*P*=0.318). With regard to the comparison of toricity, the number of eyes that showed 1 and 2 scale toricity differences between each device pair was as follows: 46 (45.5%) and 3 (3.0%) eyes, respectively, between iTrace and IOLMaster; 41 eyes (40.6%) and 1 eye (1.0%), respectively, between iTrace and Pentacam; and 49 (48.5%) and 5 (5.0%) cases, respectively, between IOLMaster and Pentacam (Figure 3).

## 4. Discussion

As demonstrated by previous studies, we found significant differences in mean keratometry, astigmatism magnitude, and steep keratometry data obtained by IOLMaster and Pentacam [12, 13]. Moreover, IOLMaster obtained higher corneal keratometry values than did iTrace. Differences in the corneal keratometry values obtained by the three devices can be attributed to differences in the measured corneal diameter and measuring technologies [9]. Moreover, IOLMaster 500 device provides higher steep keratometry values than do the other two devices; this may be because the former has the smallest measuring diameter than do the other two devices [14].

The Barrett toric calculator considers the posterior corneal curvature, lens position, and the thickness and shape of the lens [8]. Moreover, this calculator employs the Universal II formula, which predicts the IOL power to estimate the IOL position and use that to calculate the effect of IOL cylinder power at the corneal plane. According to a previous study, it performs better than other calculators [7]. Therefore, we used this free online calculator to test the influence and compatibility of different corneal keratometry measurement devices in this study.

Using the Barrett toric calculator, we found significant differences in IOL power between every device pair. However, because the most common interval of IOL was 0.5 D, differences between iTrace and IOLMaster and between iTrace and Pentacam should be noted clinically. Moreover, around 20% of cases demonstrated an IOL power difference ≥1.0 D between iTrace and IOLMaster and between iTrace and Pentacam. The IOL power difference between IOLMaster and Pentacam was the smallest, and it was within the range of ±0.5 D in 94.1% of the cases. The toric IOL interval is 0.5–0.75 D (T2–T9) for each scale. Regarding toricity, 49 (48.5%), 42 (41.6%), and 54 (53.5%) cases showed more than 1 scale difference between iTrace and IOLMaster, iTrace and Pentacam, and IOLMaster and Pentacam pairs, respectively. Moreover, 39 (38.6%), 49 (48.5%), and 45 (44.6%) cases showed both IOL power of within ±0.5 D and same toricity between iTrace and IOLMaster, between iTrace and Pentacam, and between IOLMaster and Pentacam, respectively. Our findings show that a big proportion of cases (more than a half) demonstrated an inconsistent result. These findings also emphasize that ophthalmologists should be careful while planning cataract surgery on the basis of toricity, especially when the measured data and calculated results are inconsistent among different devices. No significant difference was found for the toricity axis among the three groups. Therefore, the overcorrection and undercorrection of astigmatism magnitude are the main problem during the surgical planning.

A limitation of this study was that we included no subgroups showing differences in with-the-rule, against-the-rule, and oblique corneal astigmatism calculations. The online Barrett toric calculator considered the posterior corneal astigmatism as predicted or measured type in the toricity calculation. If the eyes are grouped into different subgroups, the results may differ. Moreover, there are no postoperative data to demonstrate which device measured a more precise value in a clinical setting. This should be investigated in future studies. However, the differences identified in this study are helpful for ophthalmologists for planning surgery in patients with astigmatism [15].

In conclusion, the present study evaluated the comparability of toric IOL power and toricity calculated on the basis of the data obtained from three different devices using the online Barrett toric calculator. The corresponding differences should be noted in clinical practice.

## Figures and Tables

**Table 1:** Patient demographics.

Characteristic	Patients (*n* = 101)
Eyes (% right eyes)	55 (54.5%)
Age, y (mean ± SD)	66 ± 10
Sex (% male)	56 (55.4%)
Axial length, mm (mean ± SD)	23.37 ± 0.91
Anterior chamber depth, mm (mean ± SD)	3.10 ± 0.37
With-the-rule astigmatism (% eyes)^*∗*^	29 (28.7%)
Against-the-rule astigmatism (% eyes)^*∗*^	65 (64.4%)
Oblique astigmatism (% eyes)^*∗*^	7 (6.9%)

SD: standard deviation; ^*∗*^corneal astigmatism measured with IOLMaster 500.

**Table 2:** Mean anterior corneal keratometry values obtained using iTrace, IOLMaster, and Pentacam.

	iTrace at 3 mm	IOLMaster at 2.3 mm	Pentacam at 3 mm
	(*n* = 101)	(*n* = 101)	(Centered on the corneal apex) (*n* = 101)
Astigmatism magnitude (D)	1.86 ± 0.77	1.95 ± 0.87	1.83 ± 0.79
	Range: 0.61–4.23	Range: 0.34–4.50	Range: 0.40–3.80
Astigmatism axis (degree)	97 ± 68	99 ± 64	94 ± 47
	Range: 0–179	Range: 1–179	Range: 0–179
Flat keratometry (D)	43.69 ± 1.58	44.04 ± 1.61	44.01 ± 1.58
	Range: 39.58–47.74	Range: 39.57–47.74	Range: 39.40–47.60
Steep keratometry (D)	45.55 ± 1.62	45.99 ± 1.57	45.84 ± 1.60
	Range: 41.02–48.77	Range: 42.13–49.41	Range: 41.10–49.10
Mean keratometry (D)	44.62 ± 1.55	45.02 ± 1.53	44.92 ± 1.54
	Range: 40.30–48.26	Range: 40.85–48.51	Range: 40.25–48.25

D: diopter.

**Table 3:** Mean difference between anterior corneal keratometry values for each pair of devices.

	iTrace-IOLMaster	iTrace-Pentacam	IOLMaster-Pentacam
Astigmatism magnitude (D)	−0.10 ± 0.33	0.03 ± 0.27	0.12 ± 0.38
	*P* = 0.005	*P* = 0.325	*P* = 0.002
Flat keratometry (D)	−0.35 ± 0.27	−0.31 ± 0.33	0.04 ± 0.36
	*P* < 0.001	*P* < 0.001	*P* = 0.310
Steep keratometry (D)	−0.45 ± 0.26	−0.29 ± 0.30	0.16 ± 0.24
	*P* < 0.001	*P* < 0.001	*P* < 0.001
Mean keratometry (D)	−0.40 ± 0.20	−0.30 ± 0.29	0.10 ± 0.24
	*P* < 0.001	*P* < 0.001	*P* < 0.001

D: diopter.

**Table 4:** Intraocular lens power and toricity calculated using the online Barrett toric calculator for each device.

	iTrace *n* = 101	IOLMaster *n* = 101	Pentacam *n* = 101
IOL power (D)	20.8 ± 2.8	20.3 ± 2.9	20.4 ± 2.8
	Range: 12.5–27.0	Range: 12.5–27.0	Range: 12.5–27.0
Toricity (T)	5 ± 2	5 ± 2	5 ± 2
	Range: 2–9	Range: 2–9	Range: 2–9
Axis (degree)	62 ± 66	59 ± 64	64 ± 65
	Range: 1–180	Range: 0–179	Range: 1–179

D*:* diopter.

## Data Availability

The raw datasets supporting the conclusions of this article are available from the corresponding author Dr. Xiaogang Wang upon request (e-mail: movie6521@163.com).
